# Systematic development of a patient-reported ONCOlogical-ROUTinE-Screening (ONCO-ROUTES) procedure at the University Cancer Center Regensburg

**DOI:** 10.1007/s00432-024-05955-4

**Published:** 2024-09-28

**Authors:** Julia Maurer, Anna Saibold, Katharina Gerl, Michael Koller, Oliver Koelbl, Tobias Pukrop, Sandra Windschuettl, Sabine Einhell, Anne Herrmann-Johns, Georgios Raptis, Karolina Mueller

**Affiliations:** 1 University Cancer Center Regensburg, University Hospital Regensburg, Regensburg, Germany; 2 Bavarian Cancer Research Center (BZKF), Regensburg, Germany; 3 Department of Information Technology, University Hospital Regensburg, Regensburg, Germany; 4 eHealth Laboratory, OTH Regensburg, Regensburg, Germany; 5 Center for Clinical Studies, University Hospital Regensburg, Regensburg, Germany; 6 Department of Radiation Oncology, University Hospital Regensburg, Regensburg, Germany; 7 Department of Internal Medicine 3, University Hospital Regensburg, Regensburg, Germany; 8 Department for Epidemiology and Preventive Medicine, Medical Sociology, University Regensburg, Regensburg, Germany

**Keywords:** Patient-reported outcomes (PROs), Digitalization, Routine screening, Interoperability, Supportive therapy

## Abstract

**Purpose:**

The evaluation of treatment success and progression in oncology patient-reported outcomes (PROs) is playing an increasingly important role. Meanwhile, PROs are a component of the certification requirements of the German Cancer Society for oncology centers. PROs are used to provide supportive therapy. There is currently no instrument that fully covers the requirements. At the University Hospital Regensburg (UKR), a digital ONCOlogical-ROUTinE-Screening (ONCO-ROUTES) procedure was developed in order to assess the need for supportive therapy in a standardized way and to provide patients with supportive interventions tailored to their needs.

**Methods:**

On the basis of current requirements and guidelines, the development of ONCO-ROUTES was supported by experts in focus groups and interviews, and digitalization was carried out in connection with the IT infrastructure.

**Results:**

A Needs-based, Quality-of-life (QoL) and Symptoms Screening (NQS^2^) tool already established in the routine at the UKR was further developed into ONCO-ROUTES, which is made up of the domains therapy phase, nutrition, tobacco use, alcohol use, quality of life, general condition/functional status, physical activity, psychooncology, social services, and further support needs. By linking the digitized questionnaire to the hospital information system, the results are available for immediate use in routine operations and thus for the referral of patients for further supportive therapy.

**Conclusion:**

The digital PRO application ONCO-ROUTES is designed to involve patients in monitoring additional supportive needs and thus, improves supportive interdisciplinary treatment.

## Purpose

Patient-reported outcomes (PROs) are important in the comprehensive, holistic treatment of cancer patients to determine the need for supportive interventions. The systematic screening of patient needs is a crucial step in patient-centered treatment (Richardson et al. 2007; Singer et al. 2020).

Many clinical studies have shown further benefits of PROs such as the potential to improve outcome (Klinkhammer-Schalke et al. 2012, 2020; Basch et al. 2022) and the communication between patient and physician, and to be a decisive factor in treatment decisions when several alternative therapies are available to the patient (Laviana et al. 2020; Bartlett et al. 2021; Blood et al. 2021; Knapp et al. 2021; Del Rosario García et al. 2022; Nordhausen et al. 2022; Hilser et al. 2023; Wilson et al. 2024). This active role of patients (“shared decision-making”) has been shown to improve satisfaction and quality of life (QoL) (Baratelli et al. 2019; Absolom et al. 2021).

Therefore, in Germany, the assessment of PROs is subject to regulatory requirements based on the guidelines of the German Cancer Society (DKG) (Zertifizierung der Deutschen Krebsgesellschaft: Dokumente | DKG 2024), the German Cancer Aid (DKH) (Leitlinienprogramm Onkologie 2024) and the Association of the Scientific Medical Societies e.V. (AWMF) (AWMF Leitlinienregister 2024). Supportive interventions should be tailored to patients’ needs. A prerequisite for standardized and time-efficient assessment of patients’ needs is appropriate screening.

Standardized digital recording has significant advantages such as the transparent documentation of results and the availability of data in a timely and structured manner for evaluation in patient care with as little personnel effort as possible using a smartphone or tablet. Other advantages are filling data gaps, supporting clinical decision-making, providing access for all clinics involved in the treatment, and avoiding redundant records (Bartlett et al. 2021; Blood et al. 2021; Knapp et al. 2021; Maguire et al. 2021; Del Rosario García et al. 2022). Nevertheless, it is important to consider the accessibility of individual patient groups and their specific needs as well as their reservations about digital solutions, in order to increase acceptance and consequently improve the quality of data and thus also the quality of care (Slade et al. 2021; Del Rosario García et al. 2022; Nielsen et al. 2022). Recurring recordings over a defined period of time can reveal changes in patients’ needs and thus play a crucial role in the planning of further therapy and supportive interventions.

The objective of our project was to develop a structured, interdisciplinary, ONCOlogical-ROUTinE-Screening (ONCO-ROUTES) procedure for adult patients based on the requirements of oncological treatment and existing structures at the University Hospital Regensburg (UKR) and to establish a digital link of the screening data to the clinical patient data that are available in the hospital information system (HIS) and the tumor registry.

## Methods

This project is based on an existing ethics vote of the Ethics Committee of the University Regensburg (No. 20-1888-101).

**Fig. 1:**
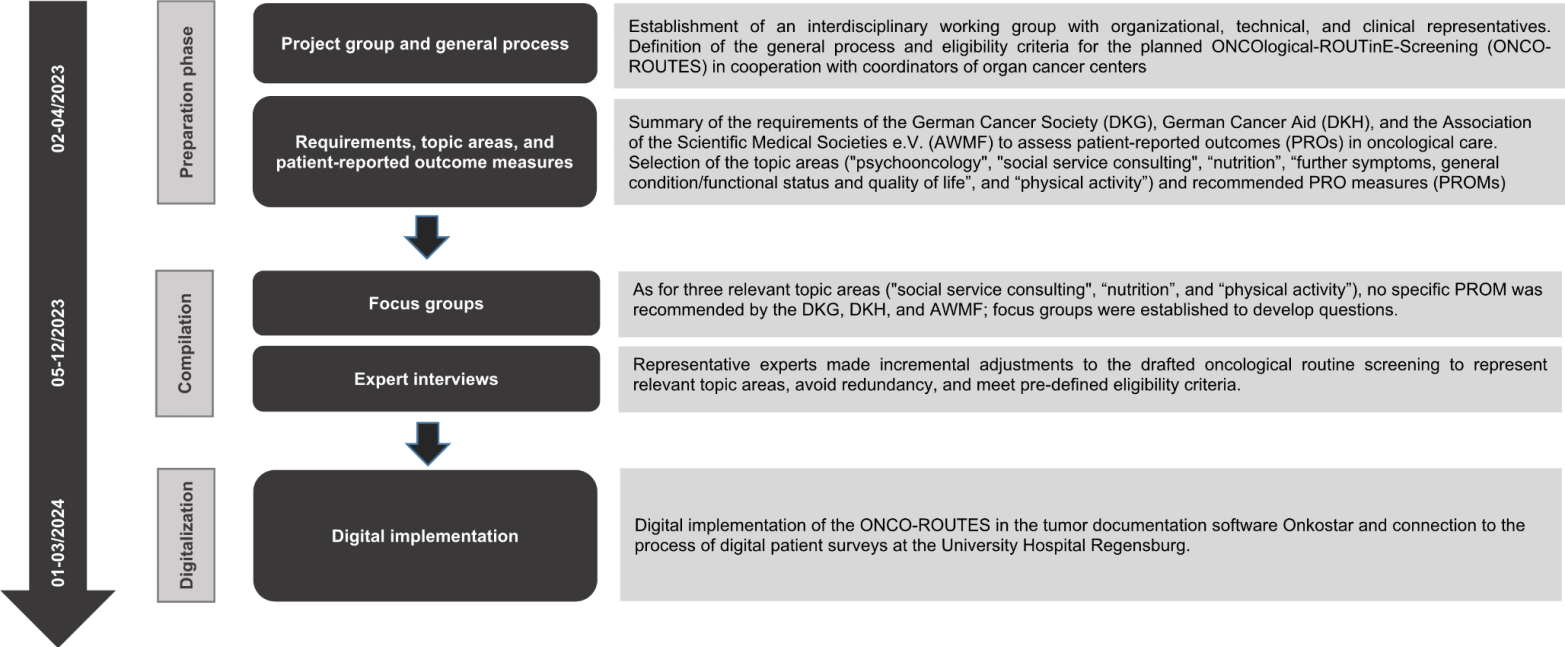
Project plan - Development of (ONCO-ROUTES)

The development and digitalization of ONCO-ROUTES at UKR lasted from 02/2023 to 03/2024. The project plan consists of three phases: preparation, compilation, and digitalization (see Fig. 1).

### Preparation

**Fig. 2:**
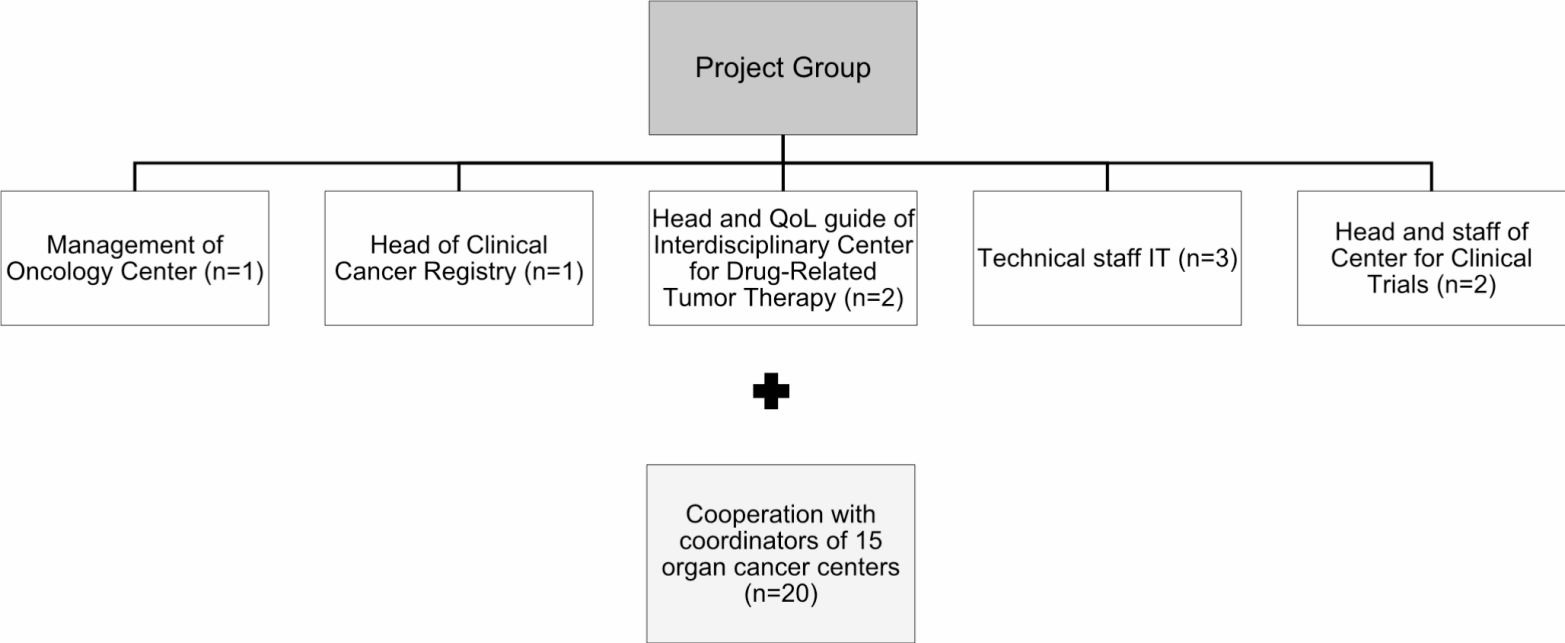
Organigram project group. In coordination with the management of the Oncology Center a project group consisting of representatives with organizational, clinical, technical, and methodological expertise was established. In cooperation with the coordinators of all the 15 individual organ cancer centers, the general process for the development and digitalization of ONCO-ROUTES in daily clinical practice as well as content related aspects of ONCO-ROUTES were defined

The preparation phase took place from February to April 2023. In coordination with the head of the Oncology Center a project group consisting of representatives with organizational and clinical (management of Oncology Center, head of Clinical Cancer Registry, head and QoL guide of Interdisciplinary Center for Drug-Related Tumor Therapy (ICT)), technical (staff IT UKR), and methodological expertise (head and staff of Center for Clinical Trials) was established (see Fig. 2).

The project group fulfilled the following tasks in the preparation phase:

First, the project group, in cooperation with the coordinators of all the 15 individual organ cancer centers at UKR, defined the general process for the development and digitalization of ONCO-ROUTES in daily clinical practice.

Second, eligibility criteria were defined by the project group in Delphi rounds and in consultation with management of the Oncology Center focusing on important aspects for usage in clinical routine.

Third, relevant topic areas were identified based on the requirements and guidelines of the DKG, DKH, and AWMF (German-S3-Guidelines for psychooncology, palliative, supportive and geriatric medicine) as well as on already established processes at the UKR. Moreover, PRO measures (PROMs) recommended by guidelines and used at the UKR were searched within the relevant topic areas.

#### Compilation of ONCO-ROUTES

Based on the findings from the preparation phase, focus groups and expert interviews were used to finalize ONCO-ROUTES between May and December 2023. Moreover, the frequency in which ONCO-ROUTES should be carried out was defined.

Regulatory requirements used in oncology and an already existing questionnaire in clinical routine at the UKR were used as a starting point for the compilation of ONCO-ROUTES.

A focus group (Bortz and Döring 2006) was established because no specific PROMs were recommended by the AWMF-S3 guidelines for the three identified relevant topic areas (psychooncology, palliative, supportive and geriatric medicine). The focus group consisted of nine health care experts from the fields of oncology, social services, nutritional counseling, physiotherapy, and a representative of the patient advisory board of the Comprehensive Cancer Centre East Bavaria (CCCO); meetings took place face-to-face. Based on a nominal group process (Delbecq et al. 1975), the focus group identified key issues to be addressed by ONCO-ROUTES in these three specific areas. In the second round, these topics were assessed according to their relevance. For topics with a relevance rating of ≥ 70%, questions for ONCO-ROUTES were derived by the project group. Finally, representatives of the focus groups finalized the prepared questions.

Several rounds of separate interviews were conducted with representative experts and members of the project group. A first draft of ONCO-ROUTES was compiled based on results of the preparation phase and focus groups. The Delphi method (Gracht 2012) was used to adapt the compiled ONCO-ROUTES. In each round, ONCO-ROUTES was reviewed to ensure that (1) each relevant topic area was adequately represented (e.g. by recommended PROM), (2) relevant topics were not missing, (3) redundancies were avoided, and (4) predefined eligibility criteria were met.

### Digitalization of ONCO-ROUTES

Between January and March 2024, the final ONCO-ROUTES procedure was subsequently digitalized. The technical process was linked to the existing software landscape of the HIS and the University Clinical Cancer Registry at the UKR. Therefore, an already developed digitalization strategy for the clinical routine was used (currently in publication process, published abstract available (Maurer et al. 2023).

## Results

### Preparation

#### Eligibility criteria

Based on the guidelines and the results of the focus group and interviews the following eligibility criteria for PROMs were defined by the project group:

ONCO-ROUTES should

- identify those patients in clinical care who need additional supportive therapy in specific areas.
- monitor QoL throughout the treatment and follow-up period.
- be used repeatedly to cover important stages in the course of treatment.
- identify changes in patient needs for all cancer patients without limitations such as specific tumor entities, severity of illness, and age.
- be easy to use and interpret.
- cover all relevant areas.
- not be excessively long.
- identify further additional support needs (specific subsequent detailed questionnaires should be administered by specialist staff in the individual supporting areas).
- be able to be answered by patients without major help of medical staff.
- be easily digitalized (a digital assessment is preferred as PRO data can be automatically stored together with clinical data).
- be automatically presented with its results in the HIS and be accessible for different interdisciplinary centers to ensure the objectives (1) avoidance of redundant measurement repetitions in different centers during an appointment or a short period of time, (2) faster and standardized assessment of the need for additional support, and (3) faster and transparent referral to supporting areas.
- be free of charge for the use of validated questionnaires due to the planned application in the routine operation of the entire oncology center.

### Relevant topics and recommended / used PROMs

The following eight relevant topic areas were found in requirements and guidelines of the DKG, DKH, and AWMF (Leitlinienprogramm Onkologie 2024; AWMF Leitlinienregister 2024; Zertifizierung der Deutschen Krebsgesellschaft: Dokumente | DKG 2024): (1) palliative medicine, (2) psychooncology, (3) social service consulting, (4) nutrition, (5) further symptoms, general condition, functional status, QoL, (6) physical activity, (7) family history, and (8) geriatric medicine. Table 1 shows the relevant topic areas along with the identified PROMs recommended in guidelines and used at the UKR. The adaptations of the DKG requirements in 12/23 were also considered.

**Table 1:** Overview of the requirements and guidelines of the DKG, DKH, and AWMF and used PROMs at the UKR

Topic area	Recommended PROMs			Used PROMs at UKR
	DKG (03/23)	DKG (adapted 12/23)	DKH/AWMF	
Palliative medicine	***MIDOS/IPOS*** should be used in neurooncological, lung cancer, and hematooncological centers	No change	***MIDOS/IPOS***	***IPOS*** in neurooncological, lung cancer, and hemato-oncological centers
Psychooncology	Screening recommended, no PROM specified	No change	***e.g. DT*** supplemented by additional psychooncological consultation request (other PROMs listed: HADS, FBK, PHQ-9, GAD-7)	***DT*** supplemented by additional psychooncological consultation request (all organ centers)
Social service consulting	No screening recommended	Screening recommended, no PROM specified	No screening	***NQS²*** (need for support in dealing with health insurance and authorities)
Nutrition	***NRS*** should be used in visceraloncological center	***e.g. NRS*** should be used in all organ centers (no other PROMs listed)	No PROM specified	***NRS*** (visceraloncological center)
Further symptoms, general condition / functional status, quality of life	No screening recommended	***e.g. MIDOS/IPOS*** (no other PROMs listed)	Screening recommended for interdisciplinary important symptoms (xerostomia, mucositis, diarrhea, changes in smell or taste and polyneuropathy) (e.g. ***PRO-CTCAE***)	***NQS² (EORTC QLQ-C15-PAL***, ***modified KPSS***, ***physical general state)***
Physical activity	No screening recommended	Screening recommended, no PROM specified	No screening recommended	***NQS²*** (Need to participate in sports and exercise opportunities)
Family history	***DKG-specific questionnaires*** should be used for colorectal cancer, breast and/or ovarian cancer, sarcoma, and renal cancer	No change	Recommended entity specific in individual guidelines for colorectal cancer, breast and/or ovarian cancer, and renal cancer	***DKG-specific questionnaires*** used for colorectal cancer, breast and/or ovarian cancer, sarcoma, and renal cancer
Geriatric medicine	No screening recommended	***e.g. PRO-G8*** for oncological patients ≥ 70 years (no other PROMs listed)	***PRO-G8*** for oncological patients ≥ 70 years	No PROM used, implementation planned

DKG - German Cancer Society; DKH - German Cancer Aid; AWMF - Association of the Scientific Medical Societies e.V.; UKR - University Hospital Regensburg; PROMs – patient-reported outcome measures; MIDOS - Minimal Documentation System for Distressing Symptoms; modified KPSS – Karnofsky Performance Status Scale; IPOS - Integrated Palliative Outcome Scale; DT - Distress Thermometer; HADS - Hospital Anxiety and Depression Scale; FBK - Fragebogen zur Belastung von Krebspatienten; PHQ-9 - Patient Health Questionnaire – Depression Modul; GAD-7 - Generalized Anxiety Disorder Scale-7; NQS² - Needs-based, Quality-of-life and Symptoms Screening

NRS - Nutritional Risk Screening; QLQ-C15-PAL - EORTC Quality of Life Questionnaire Core 15 Palliative Care; PRO-CTCAE; PRO-G8

In palliative medicine, the Minimal Documentation System for Distressing Symptoms (MIDOS) (Plöger 2016) or the Integrated Palliative Outcome Scale (IPOS) (Dugas 2024) have been recommended by the DKG, DKH, and AWMF especially for neurology, lung cancer, and hematooncology centers (Zertifizierung der Deutschen Krebsgesellschaft: Dokumente | DKG 2024; Leitlinienprogramm Onkologie: Palliativmedizin 2024). Both questionnaires consist of an assessment of symptoms such as pain, vomiting, shortness of breath, fatigue, depression, and anxiety. However, these symptoms could also be stratified to the relevant topic area “further symptoms, general condition, functional status, quality of life”. The IPOS is already in use at the UKR in the recommended centers. According to the experts at the UKR, the IPOS is better suited to record a patient’s individual symptoms. It has already been digitally connected to the tumor documentation software. The pilot project and the established digitalization strategy are currently under review.

In psychooncology screening, the Distress Thermometer (DT) (Mehnert et al. 2006) and an additional question about the need for counseling are required by the AWMF-S3 guideline (Leitlinienprogramm Onkologie: Psychoonkologie 2024). The DT is used at the UKR in all organ centers of the Oncology Center.

Moreover, ae PROM on patient needs, QoL, and symptoms named NQS^2^ was developed by an interdisciplinary team at the UKR for the routine of an interdisciplinary cancer outpatient clinic ICT (Windschüttl et al. 2021). This questionnaire assesses the (1) current therapy phase, (2) general condition using the modified Karnofsky performance status scale (KPSS), (3) general physical condition, (4) QoL using the EORTC Quality of Life Questionnaire Core 15 Palliative Care (QLQ-C15-PAL (EORTC - Quality of Life 2018), and the 4) need for support and information based on the SCNS-SF34 (Sklenarova et al. 2015). With this questionnaire, deficits in thecounseling and referral of professional support and care services can be identified, especially in the intersectoral and multiprofessional outpatient care of cancer patients.

Besides the need for psychooncological support, the NQS² also covers the need for social service support. No specific screenings for social service consulting are recommended by DKG, DKH, and AWMF.

For nutritional screening, the Nutritional Risk Screening (NRS) form (Kondrup et al. 2003) is prescribed as standard in the DKG and DKH guidelines. Some organ centers of visceral oncology at the UKR use the NRS in clinical routine.

Screenings for other symptoms, general condition, functional status, and QoL were not specified by the DKG in 03/12, but the requirements were adapted in 12/23 with the recommendation to use MIDOS or IPOS (Zertifizierung der Deutschen Krebsgesellschaft: Dokumente | DKG 2024). The AWMF S3 guideline on supportive therapy in oncology (Leitlinienprogramm Onkologie: Supportive Therapie 2024) recommends to additionally record the interdisciplinary important symptoms of dry mouth, mucositis, polyneuropathy, diarrhea, and changes in smell or taste (e.g. in the form of PRO-CTCAE (Common Terminology Criteria for Adverse Events) (Patient-Reported Outcomes version of the Common Terminology Criteria for Adverse Events (PRO-CTCAE) 2024). At the UKR (ICT), QoL is covered by the developed NQS².

Moreover, this questionnaire NQS² covers the willingness to participate in exercises. The DKG, DKH, and AWMF do not recommend any specific PROM for physical activity screening.

On the subject of family history, the DKG provides hospitals with a selection of questionnaires. The completion is mandatory for patients diagnosed with colorectal cancer, breast and/or ovarian cancer, sarcoma, or renal tumor in the case of increased genetic risk for special entities (Zertifizierung der Deutschen Krebsgesellschaft: Dokumente | DKG 2024). These questionnaires are used at the UKR.

The geriatrics guideline (AWMF Leitlinienregister 2024) also recommends geriatric screening for patients aged 70 years and older. The DKH and the AWMF recommend the PRO-G8 (Soubeyran et al. 2011). To date, no comprehensive geriatric assessment has been established at the UKR. Based on the updated certification requirements, this is being planned separately from ONCO-ROUTES.

Based on the predefined eligibility criteria, the following areas were considered relevant: “psychooncology”, “social service counseling”, “nutrition”, “further symptoms, general condition/functional status and quality of life”, and “physical activity” (Table 1).

The areas “palliative medicine”, “family history”, and “geriatric medicine” were not included for two reasons. First, these areas are only relevant for a specific group of patients. Second, separate specialized screenings within these areas are already established or in the process of being implemented at the UKR. In addition, the IPOS has a high redundancy to the PROM, which is used for the topic area “further symptoms, general condition / functional status, quality of life”.

#### Compilation of ONCO-ROUTES

##### Psychooncology

The recommended DT is a short, validated instrument independent of tumor entity and stage for patients’ self-assessment and consists of two parts (Broekmans 2020). In the first part, patient rate their stress on a visual analogue scale (VAS) from 0 (“Not stressed at all”) to 10 (“Extremely stressed”). The cut-off for psychooncological consultation is ≥ 5. The second part is divided into five domains with a total of 36 decision items, including practical, family, emotional, spiritual, and physical problems (Mehnert et al. 2006).

According to the experts, there was no advantage to using any of the other PROMs (HADS (Petermann 2011), FBK (Herschbach et al. 2004; Herschbach 2021), PHQ-9 (Whitney et al. 2010), GAD-7 (Spitzer et al. 2006) (Leitlinienprogramm Onkologie: Psychoonkologie 2024) alternatively recommended in the AWMF-S3 guideline. Moreover, the most aspects of the other PROMs are also covered by other questions in the compiled screening. The project group decided to only include the DT VAS to avoid lengthening and redundancy. In addition, questions were added about the patient’s wish for psychooncological counseling and if there is a person available for support. After a need for support is identified, the second part of the DT should only be administered by a psychooncologist.

### Social service consulting

Guidelines recommend needs assessment and advice from social services without specifying a particular screening. According to the German Association for Social Work in Health Care (DVSG), checklists are recommended for assessing the need for a social service. As a minimum, the following information should be obtained: age, previous illnesses, social situation, previous independent or dependent care situation, assistance with personal hygiene, nutrition, excretion, mobility, and taking medication (DVSG: Entlassmanagement 2024).

Based on the question included in the NQS² and the focus group, the following aspects were included in ONCO-ROUTES: no person available for support in daily life, dispositions, further need for counseling in various areas (e.g., finances, insurance), and desire for rehabilitation. Each question can trigger a consultation with social services.

### Nutrition

The Nutritional Risk Screening (NRS) (Kondrup et al. 2003) developed by ESPEN in 2002 is recommended for inpatient hospital stays. It is divided into a pre-screening, which includes questions about body mass index, weight loss in the past 3 months, and reduced food intake in the past week, presence of a serious illness, and a main screening (Schöneberger et al. 2022). If nutritional risk is identified, a nutrition plan is drawn up and/or the screening is repeated during the course of therapy. As the NRS is assessed by third parties and not by the patients themselves, it is problematic for inclusion. For the same reason, some other tools, such as the Malnutrition Universal Screening Tool (MUST) (Schütz 2005), cannot be included in the selection.

The focus group identified 26 key issues (16 of which were classified as eligible for recording). However, most of these issues were considered by the experts to be too specific for inclusion and should rather be queried and assessed in direct consultation with specialists. Therefore, the project group decided to include a self-developed question, adapted from the pre-screening of the NRS, regarding unintentional weight loss in the past three months, which acts as a trigger for consultation.

In addition to ONCO-ROUTES, a complete recording of the NRS is still necessary for selected entities (especially in the visceral oncology center) and is carried out as part of the nutrition consultation at the UKR.

Further symptoms, general condition/functional status, and QoL.

The DKG recommends specific PRO questions regarding further symptoms, general condition/functional status, and QoL by using, for example, the MIDOS or IPOS (Zertifizierung der Deutschen Krebsgesellschaft: Dokumente | DKG 2024). The AWMF-S3 guideline for supportive therapy (Leitlinienprogramm Onkologie: Supportive Therapie 2024) recommends the screening for the interdisciplinary important symptoms (xerostomia, mucositis, diarrhea, changes in smell or taste, and polyneuropathy) (e.g. using PRO-CTCAE).

In the oncology setting at the UKR, both the KPSS and Performance Status of the ECOG (Eastern Cooperative Oncology Group) (Patient-Reported Outcomes version of the Common Terminology Criteria for Adverse Events (PRO-CTCAE) 2024) are used in clinical routine. Both scales assess the physical performance status. However, neither instrument is a PROM. In NQS2, the modified KPSS and the EORTC QoL Questionnaire Core 15 Palliative Care (QLQ-C15-PAL (EORTC - Quality of Life 2018) are already in use. The questionnaires of the EORTC are well established at various centers of the UKR and are frequently used in the context of clinical studies all over the world, which leads to good comparability of the results. The QLQ-C15-PAL is used as a validated short version of the QLQ-C30. 15 items are combined into ten scores, namely quality of life, physical function, emotional function, pain, dyspnea, insomnia, appetite loss, constipation, nausea and vomiting, and fatigue.

A switch to another exemplary recommended questionnaire (such as MIDOS or IPOS) was avoided by the project group, as no benefit would be achieved due to the large number of content overlaps with the QLQ-C15-PAL. To optimize the length of the screening, QLQ-C15-PAL was preferred to the QLQ-C30 (EORTC - Quality of Life 2017).

Both, the modified KPSS and the EORTC QLQ-C15-PAL were included in ONCO-ROUTES. Due to their clinical relevance according to expert panels and the AWMF-S3 guideline (Leitlinienprogramm Onkologie: Supportive Therapie 2024), these PROMs were complemented by the following symptom items mentioned in PRO-CTCAE: xerostomia, mucositis, diarrhea, changes in smell or taste and polyneuropathy. Therefore, the response scale of the PRO-CTCAE items was adjusted to match the response scale of the QLQ-C15-PAL.

No fixed trigger leading to a specific consultation was included. An evaluation of the results of the individual items is planned during the medical round. If necessary, treatment modalities can be adjusted or specific supportive therapies can be initiated by the clinician.

### Physical activity

The main objective for this area is to record physical activity and its changes during oncological therapy and to initiate appropriate support. No suitable instrument was recommended.

The NQS² already includes a question on the desire to participate in physical activities. This question was not included directly so as not to highlight any unattainable options. Due to the limited number of therapy places at the UKR, the experts would first like to have direct patient contact in order to be able to filter out those patients who have a specific need.

31 key topics were identified in the focus group (eight of which were considered relevant for inclusion). Over several rounds, the experts agreed on four relevant questions about the current maximum physical activity (performance and hours/week), the change in physical activity in the past three months, and the expectations of exercise in therapy. A physiotherapy consultation is intended if patients (1) are not able to do physical activities, or (2) were physically active for less than 2.5 h per week, (3) reported substantial deterioration over the past three months, or (4) reported that they do not expect any improvements regarding their diagnosis/therapy through physical activity. In physiotherapy consultation, further specific questionnaires and tests will be used by specialists. Based on these results, physicians will create an individual sports program for the patient. If it is not necessary or possible to carry out the program directly at the UKR, patients are linked to programs close to home, which are arranged in cooperation with the attending family doctor.

### Further relevant areas

As part of the expert rounds, other areas were included, such as the recording of the treatment phase (as already established in the NQS²), to evaluate the results from a clinical perspective. To ensure comprehensive screening from a clinical perspective, tobacco (self-developed questions) and alcohol use (using the validated Alcohol Use Disorders Identification Test-Consumption (AUDIT C instrument) (AUDIT derivatives 2024)) were also included. These questions are not provided with specific triggers but may lead to consultations after clinical evaluation.

Finally, ONCO-ROUTES was supplemented by further defined needs for counseling and information of the ICT questionnaire. These further needs could not be integrated into the relevant key areas. Thus, a new section was created that included medical needs (e.g. desire to have children), other needs (e.g. hair loss counseling), and an option for free text entry if a specific need is not listed. As soon as one need is confirmed, a consultation follows.

### Final ONCO-ROUTES

In summary, ONCO-ROUTES is composed of the following domains: treatment phase, nutrition, tobacco use, alcohol use, quality of life, general condition/ functional status, physical activity, psychooncology, social service, and current support needs (Table 2).

**Table 2:** Final ONCOlogical-ROUTinE-Screening Tool (ONCO-ROUTES)

Area	Instrument/ Components	Number of questions	Contents	Triggers for consultation
Therapy phase	Self-developed question (***NQS²***)	1	Therapy / follow-up status	No fixed trigger for consultation
Nutrition	Modified ***NRS*** item	1	Unintentional weight loss in the past three months	Consultation with nutritional therapy in case of unintentional weight loss in the past three months
Tobacco use	Self-developed questions	3	Current and previous tobacco use	No fixed trigger for consultation. Evaluation in medical round
Alcohol use	***AUDIT-C***	3	Current and previous alcohol use	No fixed trigger for consultation. Evaluation in medical round
Further symptoms, general condition / functional status, quality of life	Modified ***KPSS*** + self-developed physical general state ***+EORTC QLQ-C15 PAL*** (***NQS²***) + additional modified symptoms from the ***PRO-CTCAE***	22	Disease-related limitations and need for help in daily life, quality of life, physical function, emotional function, and symptoms: dyspnea, pain, insomnia, fatigue, appetite loss, constipation, nausea and vomiting, xerostomia, mucositis, diarrhea, changes in smell or taste, polyneuropathy	No fixed trigger for consultation. Evaluation in medical round
Physical activity	Self-developed questions	4	• Current maximum physical activity (incl. duration/week) • Change in physical activity in the past three months • Expectations of exercise on therapy	**Consultation to physiotherapy in case of**: • Significant deterioration in physical activity in the past three months • No expectation of impact of exercise on therapy
Psychooncology	***DT VAS*** + self-developed questions	3	• Stress of the past week (thermometer) • Additional need for consultation • No person available for psychological support	**Consultation with psychooncology in case of**: • Score ≥ 5 in the thermometer • Additional need for consultation • No person available for psychological support
Social service consultation	Self-developed questions (***NQS²***)	4	• Person available for support in everyday life • Existing dispositions • Further need for counselling in various areas (e.g., finances, insurance) • Desire for rehabilitation	**Consultation with social services in case of**: • No person available for support in everyday life • Further need for counselling in various areas • Desire for rehabilitation
Current support needs	Self-developed questions (***NQS²***)	3	Further needs for medical or additional support (predefined areas and free text field)	Consultation with patient guides in case of need for support
Total number of items		**44**		

***Sequence***: Start of therapy or event of relevant changes in status during the course of the disease (progress, relapse) (at least every 3 months) and during regular follow-up

As described above, some questions were provided with specific triggers that require the patient to be referred to the corresponding specialist area. Regarding the time or sequence of the questions, the experts recommended that screening should be performed at the beginning of therapy. Furthermore, it should be repeated at least every three months during the course of therapy and also at regular follow-ups. At the discretion of the physician, the screening should be repeated if relevant changes occur in the course of the disease, such as disease progression or recurrence.

### Digitalization of ONCO-ROUTES

**Fig. 3:**
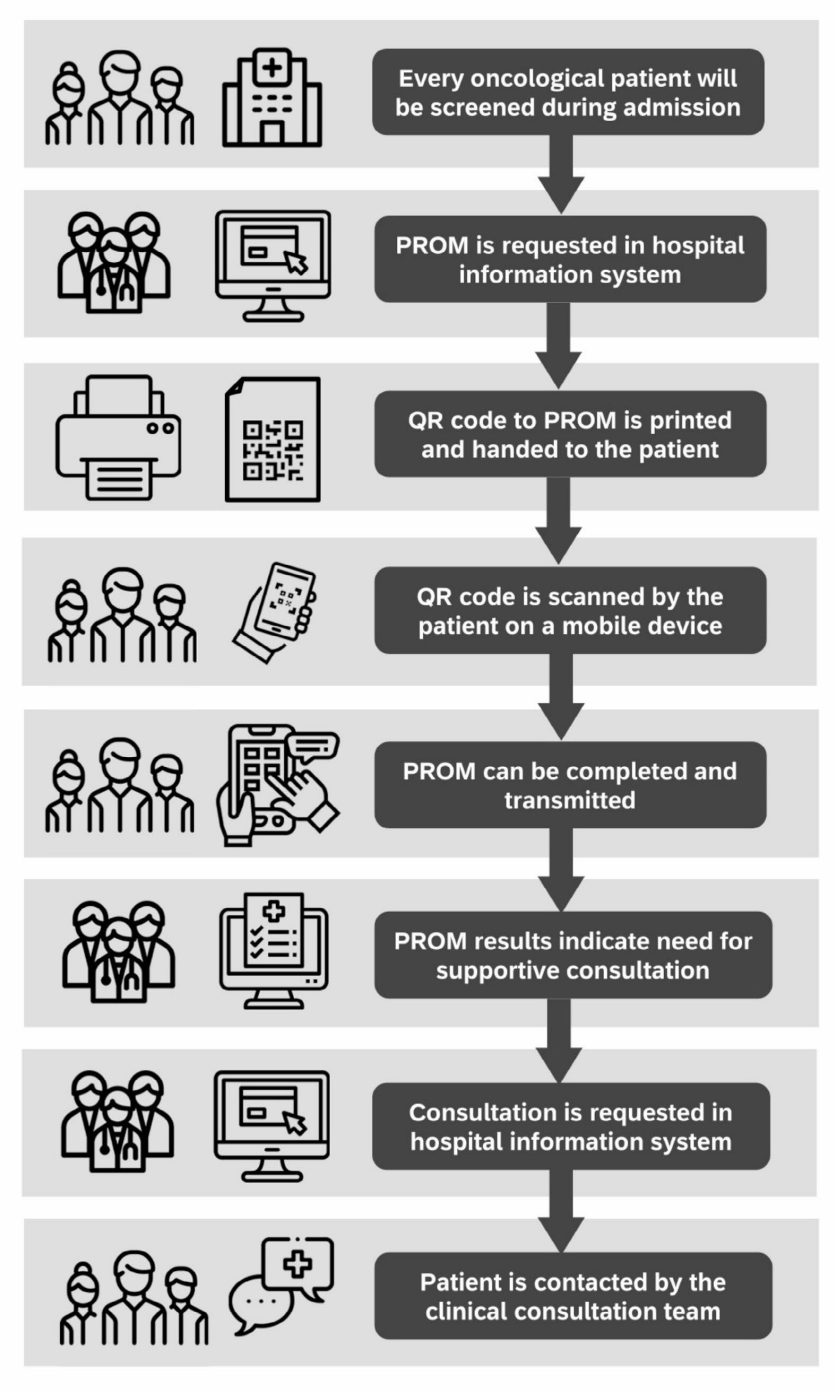
Sequence of the digital process of PROM (patient-reported outcome measures)

In the HIS, surveys can be requested by oncology departments (control centers, case management, etc.) for oncology patients. A QR code will be generated. Patients may access the digital screening by scanning the QR code via their own smartphone or a tablet provided. Figure 3 presents the sequence of the digital process.

**Fig. 4:**
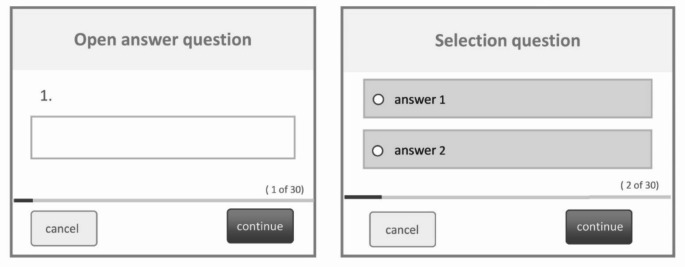
View of the digital PROM in the tumor documentation software for answering free text and decision questions

As part of the preparatory work for the digitalization of the IPOS questionnaire at the UKR, great importance was attached to a standardized, patient-friendly layout for all further patient surveys. This design was also adopted as part of this project. For a better overview and readability, only one question with all possible answers or response scales is displayed on the screen at a time. Corresponding buttons allow the user to navigate between the questions for correction or to skip individual questions if they do not wish to answer them. Mandatory fields have not been defined in order to avoid incorrect answers or the termination of the assessment. Possible question types include radio buttons, drop-down menus, or the entry of free text. Example questions are compiled in Fig. 4.

The screening was designed as a web application. For security reasons, the server is located in the demilitarized zone of the UKR, so that the separation of the HIS from the internet is still guaranteed. The digital connection between the HIS and the tumor documentation software is established via an individual identification number for each patient. In the tumor documentation software, a separate form is created in the patient record for the patients who have completed the screening, which can be filtered in the software to identify these patients. The results of the screening are stored in the tumor documentation software together with the clinical data of the patients and are directly reflected in the patient record in HIS, including the indications for consultation based on the triggers. The treating physician is responsible for further steps (commissioning of consults, adjustment of supportive therapy, etc.). The time interval between the screening and the subsequent consultation depends on the urgency of the individual supportive areas (usually within 1 week). The specialists also document the findings of the consultation in the HIS so that they can be viewed by everyone involved in the treatment (physicians, supportive teams, etc.).In case of technical difficulties, the questionnaires can still be filled out on paper and then documented in the tumor documentation software.

## Discussion

### Compilation of ONCO-ROUTES

Based on the requirements of the guidelines of the DKG, DKH, and AWMF, we aimed to develop a longitudinal, comprehensive, interdisciplinary, digital screening procedure named ONCO-ROUTES with the main aim to improve the treatment of all oncological patients.

A review of the current study situation in Germany shows a concentration on entity-specific recording of PROs for the purpose of quality development in oncological care for entities with very high incidence rates (e.g. prostate carcinoma, breast carcinoma) (Kowalski et al. 2020, 2024; Karsten et al. 2021).

A systematic and better assessment of symptoms and functional limitations due to PROMs in daily clinical practice allows for targeted interventions at all levels (somatic, psychological and social) (Fromme et al. 2004; Laugsand et al. 2010), the benefits of which have already been clearly demonstrated in several studies (Klinkhammer-Schalke et al. 2012, 2020).

This derivation of individually necessary interventions or consultations required for the patient is the focus of the work described here. For this reason, it was of particular interest to design the questionnaire in such as manner as to create an access route to all relevant multidisciplinary areas by means of a limited number of questions. Due to the large number of disciplines and stakeholders involved, it was considered crucial to take up and combine or supplement already established processes or PROMs in line with the requirements, rather than generate a completely new questionnaire. Especially in large oncology centers with many organ centers and supporting areas, we believe that this concept represents an important step towards the acceptance of the planned clinical implementation and thus in minimizing the hurdles to implementation.

These hurdles also include the large variety of possible PROMs and their prioritization or harmonization into a practicable questionnaire (Kowalski et al. 2024). For this reason, existing questions were supplemented with as few redundant questions as possible, based on the currently applicable recommendations. A simple series of individual questionnaires for each domain would make it considerably more difficult for patients to complete the questionnaire.

Thus, it was decided not to include those areas that were designed for specific oncology subpopulations (“palliative medicine”, “family history”, and “geriatric medicine”). Within these areas, separate screening by specialists is mandatory to meet the specific needs of these patient groups. Additionally, the clear longitudinal comparison of data is also crucial for planning the recording times and sequences along the patient’s care pathway during and after therapy, which was also taken into account in this work.

The establishment of a focus group proved to be a very important addition to the development of ONCO-ROUTES, particularly for those areas for which there was no clearly defined recommendation for the use of specific PROMs. With the help of relevant departmental representatives, working groups involved in direct patient treatment, and patient representatives, it was possible to obtain a comprehensive view of the content-related topic, its relevance, and the comprehensible wording of the questions. Based on the results, the relevant content could be linked to an overall screening in appropriate small groups within a short period of time.

The main hurdle was to compile the questions for ONCO-ROUTES or to reformulate them on the basis of the results of the focus group in such a way that there were no redundancies between the individual topic areas and that the questionnaire as a whole had an adequate length. The main focus in the formulation of the questions was to determine the need for patients to be referred for co-assessment/treatment in the supporting disciplines. Any follow-up screening or questioning that may be indicated can then be carried out in a subject-specific manner. If necessary, the questionnaire will be adapted (including triggers for consultation) and shortened after the evaluation of the pilot phase.

The questionnaire can also be adapted to changing requirements in the future. As described above, it should always be checked in detail whether the relevant areas should be integrated into ONCO-ROUTES or whether they should be recorded by means of separate screenings, e.g. only for a limited collective or a specific entity.

### Digitalization of ONCO-ROUTES

The increasing digitalization of the health care system opens up a multitude of new opportunities for systematic data collection and quality improvement, especially in the context of the electronic recording of PROs (Meirte et al. 2020).

In Germany, in particular, there are a number of challenges due to the very late digital development and networking of the data infrastructure in the healthcare sector, which is also subject to many legal regulations (e.g. data protection, EU Medical Devices Regulation) (Kowalski et al. 2024). However, it is precisely this necessary and, above all, rapid access to the PROs determined via the HIS that is extremely crucial in routine care due to the large number of stakeholders involved. Efforts are currently being made at many centers to overcome these hurdles. A comprehensive system does not yet exist in Germany.

Based on the aforementioned preliminary work in the context of the digitalization of PROs at the UKR, we were able to quickly realize the digital implementation as part of this project and, in particular, to network the existing data systems (tumor documentation software and HIS). In the document available in the HIS, the individual items are presented in a clear form, and defined triggers will indicate a potential need for further therapy-related action. In the subsequent pilot phase, further options will be developed to continuously improve the digital process (e.g. display options in the HIS based on a traffic light principle to indicate which patients require screening).

Limitations in the digitalization in the context of the present project are currently technical hurdles, such as the lack of graphical elements in the sense of scales in the tumor documentation software and process-related issues. The latter mainly concern access to digital patient questionnaires and screenings for all patients, regardless of their digital literacy or different origins, in order to be able to guarantee complete data collection. In this respect, the conceptualization of an easily understandable and manageable process and the linguistic adaptability of PROs play an important role (Slade et al. 2021).

The aforementioned aspects are currently also the focus of other digitalization projects at the UKR and will also be taken into account here in the further development after the completion of the pilot phase.

### Challenges for the implementation in clinical routine

The development of cross-departmental questionnaires based on PROs and digital implementation faces many other hurdles, especially in the context of implementation in clinical routine (Scheibe et al. 2020; Cheung et al. 2022).

In addition to the cost for hardware and software, the necessary personnel resources play a critical role here. In addition to patient education, providing support in the handling of data collection and, in particular, the interdisciplinary evaluation of results and the initiation of interventions, the longitudinal sequence of repeated surveys must be monitored. There is also a high demand for personnel in the IT area due to the initial programming effort, regularly required evaluations, and support during operation as well as necessary adaptations. The development of a practicable and easy-to-use procedure is enormously important, especially since there is no remuneration for the routine recording of PROs to date. It is also important to provide support and training when evaluating the results and initiating any necessary interventions in order to achieve the desired positive effects (Breidenbach et al. 2021; Sibert et al. 2021; Braulke et al. 2023; Kowalski et al. 2024).

### Summary of the advantages

The expansion of the data pool of oncology patients recorded in the tumor documentation software in a structured manner (especially taking into account the longitudinal comparison) and the digital connection to the hospital information system, which can be viewed by all departments, offers an important contribution to improving oncological patient care. Uniform routine screening in the oncology field also offers positive aspects for patients and the medical staff involved in the assessment. In addition to the aforementioned improvement in medical care, a uniform questionnaire with clearly defined questioning times or repetitions and without redundant content saves resources on both sides and thus offers enormous potential for increasing acceptance.

Moreover, scientific use of the data is possible by linking them to the recorded clinical data, which opens up significant new perspectives, especially in the field of health care research.ONCO-ROUTES will allow us to get a comprehensive overview of the patient-situation regarding all relevant areas and to analyze changes in supportive needs and consecutive therapeutic adaptions in the course of treatment.

#### Conclusion and outlook

Research has shown that the collection of PROs has positive effects on the outcome of oncology patients. ONCO-ROUTES was developed to meet regulatory requirements for patient-centered care. It includes the following components: treatment phase, nutrition, tobacco and alcohol use, general condition / functional status, quality of life, physical activity, psychooncology, social service counseling, and other current support needs.

The digitalization of ONCO-ROUTES can significantly support the collection and evaluation processes in the context of routine medical care, allowing PRO data to be linked with other clinical data from the patient’s file.

In the next step, ONCO-ROUTES will be gradually implemented into clinical routine. In this setting, the challenges of implementation in an oncology center with a large number of participating organ centers and thus clinical areas will be specifically addressed. Moreover, we will gain insights in acceptance and needed assistance of digital ONCO-ROUTES.

## Data Availability

No datasets were generated or analysed during the current study.
